# Editorial: Genetics applied to mammal conservation

**DOI:** 10.3389/fgene.2022.974631

**Published:** 2022-11-14

**Authors:** Fernando Marques Quintela, Carla Martins Lopes, Gislene Lopes Gonçalves

**Affiliations:** ^1^ Instituto Nacional de Pesquisas do Pantanal, Campo Grande, Brazil; ^2^ Departamento de Biodiversidade, Universidade Estadual Paulista (UNESP), Rio Claro, Brazil; ^3^ Universidad de Tarapacá, Facultad de Ciencias Agronómicas, Departamento de Recursos Ambientales, Arica, Chile

**Keywords:** animal genetics, Artiodactyla, biological conservation, Carnivora, Mammalia, Pilosa, Rodentia

Mammals (class Mammalia) comprise a highly diverse vertebrate group, amounting to 6,495 living species recognized to date (ASM, 2022). The impressive ecomorphological diversity of mammals is evident when we contrast a 2 g bumblebee bat (*Craseonycteris thonglongyai*) and a 150-ton blue whale (*Balaenoptera musculus*). Such diversity allowed mammals to colonize all continents and a great variety of habitats, from alpine zones to mesopelagic oceans, from hyper-arid deserts to hyper-humid tropical forests. Nonetheless, human impacts such as poaching, fragmentation of habitats, climate change, pollution, and introduction of exotic species, among others, have negative consequences on mammalian populations (Ceballos et al., 2017; Koepfli and Gooley, 2020). Of the 5,881 mammalian species globally evaluated by the International Union for the Conservation of Nature (IUCN) to date, a total of 1,333 are considered threatened (categories ‛Critically Endangered’, ‛Endangered’, and ‛Vulnerable’), which corresponds to 22.6% of all evaluated species (IUCN, 2022). In other words, nearly one in four living mammal species is threatened with extinction. Additionally, 337 species are classified by IUCN as ‘Near Threatened’ while another 840 are considered ‛Data Deficient’ for an evaluation of their conservation status.

Currently, a major threat to mammals is the contraction of habitats, which poses a decrease in the species’ range and abundance, which also reflected in the genetics of organisms. Genetics applied to mammal conservation is anchored in the principles of population genetics, linking population size to susceptibility to stochastic events. In large populations reduced to small sizes, genetic diversity tends to be rapidly lost, increasing the possibility of inbreeding, and the fixation of deleterious variants, which may reduce the individual fitness. Thus, unrevealing the amount of genetic diversity in wild populations and how it is structured along the geographical distribution of the species are key contributions to developing conservation strategies for mammals (Ortega and Maldonado, 2020). These considerations have resulted in the topic ‟Genetics Applied to Mammal Conservation”, which brings together novel examples of conservation genetics. A total of eight manuscripts covering species from South and North America, and Europe effectively demonstrate the many important insights that can be gleaned from molecular genetic data, which in turn have direct implications for to the design of conservation management strategies. These manuscripts used distinct genetic tools, from classic mitochondrial genes, microsatellite loci, and cytogenetics to the modern transcriptome-derived SNPs, dealing with aspects including intraspecific genetic diversity and population structure, demographic history, phylogeography, detection of hybridization, molecular systematics, and species delimitation.

The investigation of intraspecific genetic diversity is crucial for the detection of lineages characterized by unique evolutionary histories. Valdez and D’Elía analyzed the geographic distribution of the genetic diversity of the sigmodontine cricetid *Abrothrix hirta* using 119,226 transcriptome-derived SNP loci and found four highly well-supported lineages, which were suggested to be considered as ESUs and to be recognized at the subspecific level. Phylogeographic approaches are very useful for the detection of interspecific divergences, especially in regions that experienced marked past climatic fluctuations. Reding et al. examined the phylogeographic structure of the gray fox *Urocyon cinereoargenteus* in the United States, instigated by previous data which revealed a deep divergence between populations from western and eastern parts of the country. The phylogenetic analysis of the control region and complete mitochondrial genome corroborated the east/west phylogeographic break, revealing a secondary contact zone at the Great Plain Suture Zone. In addition, the authors found *Urocyon littoralis*, currently considered a distinct species, nested within *U. cinereoargenteus* western clade. Although the impact of habitat contraction and fragmentation is expected to be more intense in large mammals, in particular in species that present highly specialized behavior, Barragán-Ruiz et al. provide original data on the distribution of genetic variation (based on microsatellites) in the giant anteater *Myrmecophaga tridactyla* along a human-modified landscape, evidencing low genetic structure. However, the authors detected moderate levels of genetic diversity, recent bottleneck and inbreeding signatures, and a past demographic reduction, which was suggested to potentially impact this emblematic species. Similarly, Chaves et al. accessed the genetic diversity among populations of the Galapagos fur seal *Arctocephalus galapagoensis*, considered Endangered by the IUCN, along with breeding rockeries located on four islands of the Galapagos. The authors found a structure composed of at least two clusters, and indicative of gene flow between two islands, suggesting that migrations are driven by the local differences in marine productivity. Chaves et al. also detected significant heterozygosity excess in microsatellite markers in three populations, which indicates the occurrence of past bottleneck events. The manuscript of Caballero et al. analyzed the mitochondrial DNA sequencing of two manatee species (*Trichechus manatus* and *T. inunguis*) in Colombia, in locations never studied before. The results showed previously unknown genetic diversity for T. manatus and significant population differentiation probably caused by human interventions in one of the rivers sampled. The authors also demonstrated that environmental DNA analysis can be used to monitor the presence and persistence of manatees in the environment by detecting the DNA traces of these species in the water of rivers.

A key field for the conservation genetics of mammals is systematics. Reevaluation of phylogenetic relationships in the speciose genera is fundamental to constructing conservation strategies, in particular when species are vulnerable or threatened. Carnovale et al. addressed the recent evolutionary history of populations of the caviomorph rodent *Ctenomys* in the Argentinean Pampas, using mitochondrial DNA sequences, and revealed that *C. talarum* and *C. pundti* complex might be considered as the same biological species, or represent lineages going through an incipient differentiation process. Such systematic updates can be actively used in the conservation strategies for these organisms, considered vulnerable and endangered, respectively. DNA sequencing has allowed conservationists to assess previously unknown genetic information of endangered species that is essential to take actions in conservation programs, e.g., to decide locations where species should be released back into the wild. Taxonomic uncertainties represent difficulties for effective conservation action in target species. The red brocket deer *Mazama americana* has been demonstrated to comprise a polyphyletic complex of cryptic species characterized by a marked chromosomal divergence. Aiming to solve part of such a problem, Peres et al. collected a specimen in the vicinity of the region linked to the binomial *Mazama rufa* (so far a synonym of *M. americana*) and designed a neotype. Following, the authors tested the specific validity of *M. rufa* through an integrative analysis using molecular (three mitochondrial genes), chromosomal and morphological databases. The results revealed that *M. rufa* is supported as a species distinct from *M. americana sensu stricto*, and it composes a sister clade of “*M. americana*” from East Amazon. The authors also analyzed fecal DNA and used the results obtained to perform modeling of the species potential distribution. *Mazama rufa* is potentially distributed from the southern Amazon to eastern Argentina.

Hybridization and introgression were not usually listed among the major extinction threats until recently. If the process of hybridization occurs naturally, it is considered part of the evolutionary history of the species involved. However, this phenomenon deserves special attention when arising as a consequence of human actions (Rhymer and Simberloff, 1996), as it can compromise the genetic integrity and evolutionary process of parental types. The manuscript of Dziech highlights the potential threats caused by the wolf-dog hybridization events to the wild wolves populations. The gray wolf (*Canis lupus*) is a top predator species whose persistence and interactions have important effects on ecological aspects of other species’ lives and their surrounding environment. The author reviewed genetic studies using different molecular and bioinformatic tools as a source of information for the identification of wolf-dog hybrids in Europe, the introgression patterns observed, and to estimate potential causes and consequences of hybridizations. The author also discussed the importance of implementing standardized and reliable methods to identify wolf-dog hybrids before making decisions for conservation actions and population management.

What draws attention in most of the manuscripts on this topic is the survey of microsatellites and mitochondrial DNA sequences, indicating that they remain important and cost-effective markers for the analysis of genetic diversity, inbreeding status, kinship, and genetic structure in mammals, particularly in species that inhabit South America. However, genomic data will empower the delimitation of cryptic species and ESUs, redrawing the studies on populations of endangered mammal species.
